# A sticky situation: regulation and function of protein palmitoylation with a spotlight on the axon and axon initial segment

**DOI:** 10.1042/NS20210005

**Published:** 2021-10-06

**Authors:** Andrey A. Petropavlovskiy, Jordan A. Kogut, Arshia Leekha, Charlotte A. Townsend, Shaun S. Sanders

**Affiliations:** Department of Molecular and Cellular Biology, University of Guelph, 50 Stone Rd E, Guelph N1G 2W1, Ontario, Canada

**Keywords:** acyl protein thioesterase, axon, axon initial segment, palmitoyl acyltransferase, palmitoylation, S-acylation

## Abstract

In neurons, the axon and axon initial segment (AIS) are critical structures for action potential initiation and propagation. Their formation and function rely on tight compartmentalisation, a process where specific proteins are trafficked to and retained at distinct subcellular locations. One mechanism which regulates protein trafficking and association with lipid membranes is the modification of protein cysteine residues with the 16-carbon palmitic acid, known as S-acylation or palmitoylation. Palmitoylation, akin to phosphorylation, is reversible, with palmitate cycling being mediated by substrate-specific enzymes. Palmitoylation is well-known to be highly prevalent among neuronal proteins and is well studied in the context of the synapse. Comparatively, how palmitoylation regulates trafficking and clustering of axonal and AIS proteins remains less understood. This review provides an overview of the current understanding of the biochemical regulation of palmitoylation, its involvement in various neurological diseases, and the most up-to-date perspective on axonal palmitoylation. Through a palmitoylation analysis of the AIS proteome, we also report that an overwhelming proportion of AIS proteins are likely palmitoylated. Overall, our review and analysis confirm a central role for palmitoylation in the formation and function of the axon and AIS and provide a resource for further exploration of palmitoylation-dependent protein targeting to and function at the AIS.

## Introduction

Neurons are large, complex, polarised cells. Their asymmetry allows the directional flow of information received on dendrites and cell body to the axon and subsequent downstream neuron. The compartmentalisation of subcellular regions with distinct functions is critical for neuronal activity, translating into complex behaviours such as learning and memory. Proteins and organelles must be sorted and trafficked to specific sites, often over long distances, each uniquely important for neuronal signaling and function. One way in which neurons precisely regulate protein trafficking is via post-translational modifications. In recent years, S-acylation has attracted significant attention due to its reversible nature, akin to that of phosphorylation, allowing for dynamic changes in palmitoylation and, subsequently, protein localisation in response to neuronal activity or signaling. Indeed, S-acylation is the most observed protein lipid modification of neuronal proteins [1] and mutations in or loss of S-acylating enzymes *in vivo* in many cases predominantly present with neurological phenotypes [2–13].

## A primer on palmitoylation

**Figure 1:**
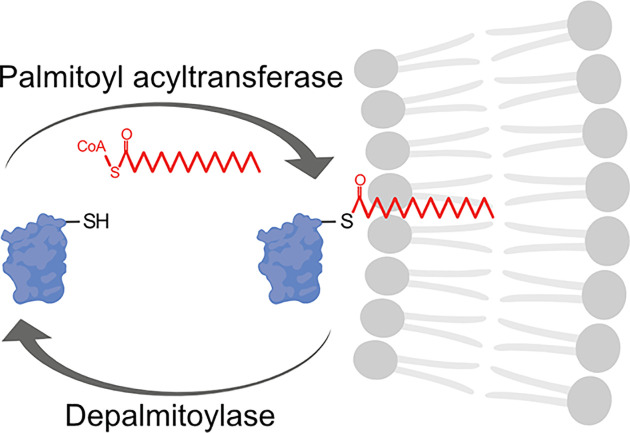
Dynamic protein palmitoylation Long-chain fatty acids (red; commonly the 16-carbon saturated palmitic acid) are added to cytosolic cysteine residues (–SH) of proteins by palmitoyl acyltransferases via a thioester bond. This increases the binding affinity of palmitoylated proteins for membranes (grey phospholipid bilayer) and/or membrane subdomains and can regulate protein–protein interactions, protein stability, other post-translational modifications, and protein function. The reverse reaction is catalyzed by depalmitoylases including acyl protein thioesterases, palmitoyl protein thioesterases, and α/β hydrolase domain-containing serine hydrolases. Figure created with BioRender.com.

S-acylation is the reversible addition of long-chain fatty acids to cysteine residues via a thioester bond (Figure 1). Saturated and unsaturated fatty acids of various lengths can be utilised, including palmitic, myristic, oleic, stearic, and arachidonic acids. However, the 16-carbon, saturated fatty acid palmitate is the most common fatty acid added to proteins, therefore, S-acylation is often referred to as S-palmitoylation or, simply, palmitoylation and will be referred to as such in this review [14–16]. More than 10% of the human proteome is predicted to be palmitoylated [17,18], including proteins involved in a diverse array of biological processes, some of which include synaptic plasticity [19,20], cardiac contractility [21], immune response [22], and pathogen–host interactions and infection [23–25].

The hydrophobic nature of fatty acids increases the binding affinity of palmitoylated proteins for membranes and can regulate protein–protein interactions, protein stability, and other post-translational modifications [26]. The localisation and function of some soluble, cytosolic proteins require palmitoylation for stable membrane association [27]. Transmembrane proteins can also be modified. Palmitoylation can alter the conformation of protein transmembrane domains or cytosolic regions, regulate protein–protein interactions, and promote association with specific membrane microdomains [28–31]. In neurons, palmitoylation is best known to regulate synaptic expression and clustering of adhesion molecules, vesicle release machinery, signaling molecules, and neurotransmitter receptors, as well as their scaffolds and kinases [32–44] (reviewed in [1,20,45–47]). Indeed, previous estimates suggest that 41% of all synaptic proteins are substrates for palmitoylation [17]. However, palmitoylation-dependent regulation of protein trafficking and targeting to other neuronal locations, particularly within the axon, is not as well understood compared with what is known at the synapse, and for this reason, will be highlighted in this review.

### Palmitoyl acyltransferases

Palmitoylation is catalyzed by the ZDHHC family of palmitoyl acyltransferases (PATs; Figure 1) that contain a conserved Asp–His–His–Cys (DHHC) active site motif within a zinc-finger cysteine-rich domain (CRD) [48]. The DHHC active site motif catalyzes palmitoylation and the CRD coordinates two zinc atoms in a zinc-finger motif, crucial for structural stability [49–53]. The PAT gene family is conserved throughout eukaryotes and the 23 mammalian ZDHHC PATs demonstrate distinct but overlapping substrate specificity [16,54].

PATs can be found throughout the endomembrane system, particularly the Golgi apparatus, endoplasmic reticulum (ER), endosomes, and plasma membrane, allowing substrates to be modified at different stages of their life [55,56]. Additionally, proteins that contain multiple palmitoylation sites can be modified at different intracellular locations by various PATs [57,58]. PATs are integral membrane proteins that contain four to six transmembrane domains with the conserved DHHC-CRD oriented to face the cytosol [16,59]. In addition to the DHHC-CRD, PATs have three other conserved motifs. The DPG (Asp–Pro–Gly) motif located amino (N)-terminal to the DHHC-CRD with unknown function. The TTxE (Thr–Thr–X–Glu, where X is any amino acid) and PaCCT (palmitoyltransferase conserved carboxyl (C)-terminus) motifs are located C-terminal to the DHHC-CRD and are critical for the enzyme’s structural integrity and activity [51,60,61]. PATs use a two-step ‘ping-pong’ mechanism to palmitoylate substrates where the cysteine residue in the DHHC motif is first autopalmitoylated and then palmitate is transferred to the substrate protein [49–51].

Despite the high similarity of the DHHC-CRD among the PATs, the N- and C-termini of these enzymes are poorly conserved, having diverse sequences and sizes contributing to substrate selectivity [16,54]. ZDHHC13 and ZDHHC17 have N-terminal ankyrin repeat domains that interact with many of their substrates’ zDHHC ankyrin-binding motifs to allow palmitoylation [62–67]. Eight of twenty-three human PATs contain a C-terminal PSD95/discs large/ZO-1 (PDZ) ligand that binds PDZ domain-containing proteins, many of which are involved in synapse formation and neuronal regulation [68]. ZDHHC14 has a C-terminal type I PDZ motif that binds its substrate the type-I PDZ domain-containing Membrane-associated Guanylate Kinase (MaGUK) family scaffold protein PSD93 (postsynaptic density 93/chapsyn 110/DLG2) [68,69]. In contrast, ZDHHC3, ZDHHC5, ZDHHC7, ZDHHC8, ZDHHC16, ZDHHC17, ZDHHC20, and ZDHHC21 have C-terminal type II PDZ motifs that have been shown to or are predicted to bind their type II domain-containing substrates [38,44,68]. Finally, the C-terminus of ZDHHC6 is unique in that it contains an Src homology 3 domain, which allows it to bind to selenoprotein K [70].

### Depalmitoylating enzymes

Due to the labile nature of the thioester bond, palmitoylation is reversible, with many proteins undergoing dynamic cycles of palmitoylation and depalmitoylation (Figure 1) [71–73]. Dynamic palmitoylation of the transferrin receptor, ankyrin, and N-RAS was first reported in the 1980s [74–76]. Even though the first depalmitoylating enzymes were discovered years before the first PAT in the 1990s [77,78], depalmitoylation is not as well understood. Acyl protein thioesterases (APTs), palmitoyl protein thioesterases (PPTs), and α/β hydrolase domain-containing proteins (ABHDs) are the three known types of depalmitoylating enzymes, all belonging to a larger family of metabolic serine hydrolases [77–85]. These three classes of depalmitoylating enzymes all contain an α/β hydrolase fold with a Ser–His–Asp catalytic triad and active serine residue consensus motif of Gly–X–Ser–X–Gly [79,82,86–90].

PPT1 was the first depalmitoylating enzyme discovered in 1993 [78] and is predominantly a lysosomal enzyme mediating depalmitoylation prior to protein degradation [91]. Mutations in the gene coding for PPT1 cause a lysosomal storage neurodegenerative disease known as infantile neuronal ceroid lipofuscinosis [92]. There is also some evidence of a cytoplasmic role for PPT1 at synapses [93–95].

APT1 (LYPLA1) and APT2 (LYPLA2) are the most well-studied cytosolic depalmitoylating enzymes, but APT1-like (APT1L; LYPLAL1) as well as the ABHD17 family (A–C) have also been shown to have cytoplasmic depalmitoylating activity [77,79,80,83,85]. ABHD10 is a mitochondrial resident depalmitoylating enzyme [82] and while APT1 has also been observed in mitochondria, its role in depalmitoylating mitochondrial proteins is unclear [96]. The consensus is that to mediate depalmitoylation of their substrates the cytoplasmic depalmitoylating enzymes need to come into proximity with the membrane. Indeed, ATP1, APT2, and ABHD17A–C have all been shown to be palmitoylated themselves [97–99] and an interesting new mechanism of membrane binding was just identified for APT2 [100]. This study from the van der Goot group showed that APT2 binds membranes in a three-step process where long-range electrostatic interactions attract APT2 to the lipid membrane. This then allows the β-tongue of APT2 to dip into the membrane, temporarily holding APT2 in place, allowing its subsequent palmitoylation by ZDHHC3 or ZDHHC7, thereby stably binding it to the membrane [100]. After APT2 identifies a target protein, it extracts palmitate from the membrane into its catalytic hydrophobic pocket for hydrolysis [100]. It will be interesting to see if the other depalmitoylating enzymes use similar mechanisms. A key outstanding question is how depalmitoylating enzymes recognise their substrates and how they are regulated by neuronal activity or signaling events.

## Palmitoylation in neurological disorders

Disrupted palmitoylation is associated with several neurological disorders in humans, including X-linked intellectual disability, bipolar disorder, schizophrenia, and neuronal ceroid lipofuscinosis (reviewed in [64,101]). Mutations in the *ZDHHC9* gene are associated with X-linked intellectual disability [5,102–105], sometimes with epilepsy [12]. Consistent with this, *Zdhhc9*-deficient mice display altered excitatory/inhibitory synapse balance, seizure-like activity [34], and altered hippocampal-based learning and memory [106].

Two closely related PATs, ZDHHC5 and ZDHHC8, have been associated with schizophrenia. Chromosomal deletions in the region including *ZDHHC5* are linked with schizophrenia as well as bipolar disorder [107,108], and a *de novo* missense mutation in *ZDHHC5* has been identified in patients with schizophrenia [109]. Significant associations between schizophrenia and small nucleotide polymorphisms in the *ZDHHC8* gene, within the schizophrenia risk 22q11 microdeletion, have been identified in multiple populations [8,110–112] but not in others [113–118]. However, consistent with a role for ZDHHC8 in schizophrenia, 22q11-deletion mice have reduced dendritic growth, spine density, and glutamatergic synapses that can be rescued with exogenous expression of ZDHHC8. Also, *Zdhhc8*-deficient mice display similar dendritic and synaptic changes and schizophrenia-like phenotypes [8,9]. Notably, a widespread reduction in palmitoylation was also observed in post-mortem dorsolateral prefrontal cortex of patients with schizophrenia, as well as reduced palmitoylation of vesicular glutamate transporter 1 (VGLUT1), RAS, and myelin basic protein [119].

Finally, mutations in genes encoding the depalmitoylating enzyme PPT1 and the palmitoylated cysteine string protein (CSP; DNAJC5) cause neuronal ceroid lipofuscinosis a lysosomal-storage neurodegenerative disease [120,121].

Evidence from animal models and cell culture systems also suggests a role for aberrant palmitoylation in various neurodegenerative diseases, including Huntington disease (HD), amyotrophic lateral sclerosis (ALS), Parkinson disease (PD), and Alzheimer disease (AD). HD is a neurodegenerative disorder caused by a CAG repeat expansion in the *HTT* gene coding for an elongated polyglutamine tract in the huntingtin (HTT) protein [122]. HTT is normally palmitoylated at Cys^214^ by ZDHHC17 and ZDHHC13 (also known as Huntingtin Interacting Proteins [HIPs] 14 and 14-like [HIP14 and HIP14L], respectively) [3,123], but mutant HTT is less palmitoylated in HD patient-derived cells and mouse models of HD [123,124]. Recent exciting findings from the Hayden and Saudou groups show that increasing palmitoylation by inhibiting the HTT depalmitoylating enzymes *in cellulo* and *in vivo* reduces cytotoxicity, mutant HTT aggregation, and restores trafficking deficits in cell culture and rescues behaviour and neuropathology in HD mice [124,125]. These two studies provide strong evidence for a role of palmitoylation in HD and rationale for targeting depalmitoylating enzymes to treat HD.

The evidence for a role of palmitoylation in other neurodegenerative diseases, including ALS, PD, and AD is not as strong but is briefly discussed here (reviewed in more depth in [64,101]). Familial ALS-linked superoxide dismutase 1 (SOD1) mutants are more palmitoylated than wildtype SOD1 in ALS-mouse models and human spinal cord. Palmitoylation prevents SOD1 disulphide bonding and, in turn, its maturation, which may contribute to the pathogenesis of ALS [126,127].

In a recently published study, Ho et al. showed that palmitoylation may play a role in PD for the first time. Inhibiting depalmitoylation reduces α-synuclein inclusions and phosphorylation at Ser^129^ (a PD neuropathological marker) as well as decreases neurotoxicity in human neuroblastoma cells, rat neurons, and induced pluripotent stem cell-derived PD patient neurons [128]. These protective effects are proposed to be due to restoring palmitoylation of microtubule-associated protein 6 (MAP6) [128]. It will be interesting to see the results of depalmitoylation inhibitor treatment in a longer term preclinical study in animal models of PD, such as was recently done in HD [125].

AD is linked to the formation of β-amyloid (Aβ) plaques derived via aberrant cleavage of the amyloid precursor protein (APP) by β-secretase (predominantly β-site APP cleaving enzyme 1 or BACE1) and γ-secretase enzymes [129]. BACE1 and γ-secretase subunits Nicastrin and APH-1 (anterior pharynx-defective 1), as well as APP itself are all palmitoylated [130–133]. While the role of BACE1 palmitoylation in pathological APP processing remains controversial (discussed in [101]), APP palmitoylation enhances amyloidogenic processing of APP to Aβ [131,134] and expressing palmitoylation defective Nicastrin and APH-1 in mice decreased amyloid deposits in the brain [135]. Additional investigations are needed to determine which palmitoylation event is the most critical for Aβ production and if targeting PATs or depalmitoylating enzymes would be a viable treatment approach.

## Palmitoylation-dependent axonal protein trafficking and targeting

While the importance of palmitoylation-dependent regulation of protein trafficking and targeting to the synapse is well established, palmitoylation-dependent protein targeting to other neuronal locations, particularly within the axon, is less well understood. Axons are long, thin projections that project up to a meter away from the cell body or soma in humans and are much longer than dendrites. The correct connection of axons to their target during development and long-term integrity of mature axons is essential for nervous system function. Indeed, axonal degeneration and axon transport deficits is a hallmark of many neurodegenerative diseases, including during acute nervous system injury [136] as well as in chronic neurodegenerative diseases such as AD, HD, and chemotherapy- and diabetes-associated neuropathies [137–139]. Thus, the efficient, precise control of protein trafficking and targeting in axons presents a significant challenge for the neuron. As with the synapse, palmitoylation of axonal proteins could provide a dynamic mechanism to control protein targeting and function. We will discuss below what is already known regarding palmitoylation-dependent protein trafficking and localisation during neuronal development and retrograde signaling from the distal axon back to the cell body or soma as well as to the cytoskeleton and presynaptic compartment. In addition, an interesting new role for palmitoylation in the regulation of protein clustering at another axonal compartment, the axon initial segment (AIS) is emerging, and we will expand on that here as well.

### Neuronal development

During neuronal development, neurite outgrowth as well as axon determination, the establishment of polarity, and axon pathfinding rely on several palmitoylated proteins (reviewed in [140]). Palmitoylation of neural cell adhesion molecule (NCAM) by ZDHHC7 directs it to lipid rafts in growth cones promoting neurite outgrowth and axon pathfinding [141–143]. Interestingly, palmitoylation of NCAM is induced by activation of the fibroblast growth factor (FGF) receptor by FGF2 treatment which stimulates neurite outgrowth in hippocampal neurons [142]. Palmitoylation of CDC42 by ZDHHC8 promotes axon growth and branching. Palmitoylated CDC42 is targeted and transported to the tip of the axon, where it modulates axon branching and length [8,144]. Rapid and dynamic palmitoylation of c-Jun N-terminal kinase (JNK) 3 is essential for axonal branching and growth during development. Unlike NCAM, palmitoylation does not regulate lipid raft association of JNK3 and instead reduces its translocation to the actin cytoskeleton to inhibit axonal branching and axon length. Thus, palmitoylation-deficient JNK3 increases axonal branching and motility of axonal filopodia [145]. Finally, GAP43 is another palmitoylated protein [146] that plays an important role in neuronal growth by modulating levels of the phospholipid phosphatidylinositol 4,5-bisphopsphate to regulate the assembly of actin-based structures and promote neurite outgrowth [147]. Targeting GAP43 to axonal membranes and to growth cones via detergent-insoluble glycolipid-enriched complexes depends on its palmitoylation [146,148,149].

Palmitoylation is also involved in the bifurcation of somatosensory afferents from the dorsal root ganglia (DRG) neuron [150]. The ligand C-type natriuretic peptide (CNP) mediates a cyclic guanosine monophosphate (cGMP)-dependent signaling pathway that promotes neurite elongation and DRG growth cone expansion. This CNP-mediated growth cone enlargement is potentiated by thioesterase inhibitors and blocked by broad-spectrum palmitoylation inhibitors. As such, cGMP-stimulated palmitoylation of proteins is thought to be implicated in axon bifurcation [150]. These findings are all consistent, demonstrating the important role of palmitoylation during neuronal development.

Several cytoskeleton proteins, molecular motors, and their scaffolds are also known to be palmitoylated and their palmitoylation plays an important role during neuronal polarisation, axon development, and maintenance. Axons and dendrites have different patterns of microtubule orientations, which is important for the development of neuronal polarity and axon versus dendritic sorting of proteins. In axons, microtubules are oriented with plus ends out whereas in dendrites microtubule orientations are mixed [151]. The stabilisation of microtubules in axons during the establishment of neuronal polarity requires the axon-enriched MAP6 [151,152]. Palmitoylation is required for MAP6 tethering to Golgi membranes and targeting of subsequent secretory vesicles to axons. Once in the axon, MAP6 is depalmitoylated by ABHD17A–C, allowing it to detach from Golgi vesicles and become enriched on microtubules within newly formed axons [152].

Furthermore, several subunits of dynein and kinesin motor proteins involved in anterograde (away from the soma) and retrograde (towards the soma) trafficking are known palmitoyl proteins or have been identified in palmitoyl proteomic studies [17,18]. Both evolutionarily conserved NUDE proteins, Nde1 and Ndel1 of the LIS1–Ndel1–Nde1 complex are palmitoylated. This complex positively regulates the retrograde motor cytoplasmic dynein. Nde1 interacts with LIS1 and dynein light and intermediate chains, and Ndel1 also interacts with LIS1 and dynein heavy and intermediate chains. Interestingly, blocking palmitoylation of Ndel1 increases its interaction with dynein as well as increases dynein activity and axonal transport [153]. Palmitoylation of dynein intermediate chain (DYNC1I1) was verified in a low throughput study [144]. DYNC1I1 plays a role in retrograde trafficking and mediates binding to its accessory factor dynactin via p150^Glued^. However, while DYNC1I1 palmitoylation was confirmed, no studies were performed to determine the role of palmitoylation in regulating DYNC1I1 function. In addition, no low throughput and functional follow-up studies have been performed on palmitoylation of other dynein and kinesin motor protein subunits identified in palmitoyl proteomic studies. Furthermore, it would be interesting to determine if subunits of the final, working motor complexes are palmitoylated and, if so, how that regulates motor function, speed, and binding to membranous cargo. Alternatively, palmitoylation may negatively regulate complex formation preventing assembly of the mature, working motor complex. These questions would be an interesting area for future investigation.

### Presynaptic protein targeting

Palmitoylation is important for the presynaptic targeting of several proteins critical for synaptic transmission. γ-aminobutyric acid (GABA) is the major inhibitory neurotransmitter in the central nervous system and is produced in GABAergic inhibitory neurons [154,155]. The GABA-synthesising enzyme glutamate decarboxylase 65 (GAD65) is a hydrophilic cytosolic protein primarily localised at presynaptic clusters within axons [156,157]. Both palmitoylation of GAD65 and the subsequent binding of GAD65 to Golgi membranes are critical for its trafficking to presynaptic clusters [158]. When GAD65 is palmitoylated, it colocalises with a small guanosine triphosphate-binding protein known as Rab5a within axonal but not with somatodendritic endosomes. In contrast, palmitoylation-deficient GAD65 is absent from presynaptic clusters despite the presence of Rab5a [156].

Several synaptic vesicle release machinery proteins are also palmitoylated. Synaptotagmin I (SYT1) is localised to synaptic vesicles where it binds Ca^2+^ ions to trigger the release of neurotransmitters from synaptic vesicles. Palmitoylation of SYT1 is required for its sorting to the presynaptic vesicle pool [144,159]. Synaptosome-associated protein of 25 kDa (SNAP25) is a target SNARE (synaptic soluble *N*-ethylmaleimide-sensitive fusion attachment protein receptor) that complexes with the vesicle SNARE vesicle-associated membrane protein (VAMP) and another target SNARE syntaxin 1. This complex mediates synaptic vesicle fusion with the plasma membrane during neurotransmitter release. While syntaxin 1 and VAMP are integral membrane proteins, SNAP25 is palmitoylated [160,161] and its palmitoylation is required for membrane binding, syntaxin-independent trafficking to the plasma membrane, and SNARE complex disassembly and exocytosis [162,163].

### Axon integrity and degeneration

**Figure 2:**
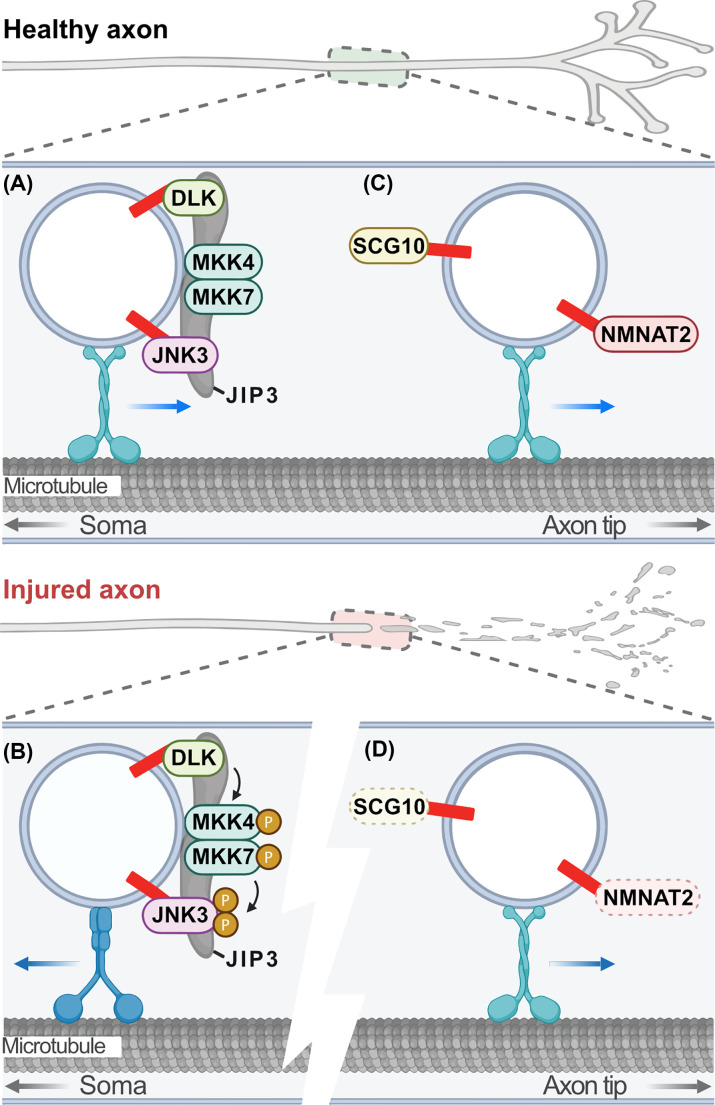
Palmitoylation-dependent regulation of axon survival and degeneration (**A**) In healthy peripheral projecting sensory or retinal ganglion axons DLK and JNK3 are trafficked on axon transport vesicles into distal axons in a palmitoylation-dependent manner along with MKK4/7 and the scaffold JIP3. (**B**) Following axonal injury DLK is activated on vesicles proximal to the injury site where it phosphorylates its direct targets MKK4/7, which then phosphorylates JNK3. This pro-degenerative signaling transport vesicle traffics retrogradely back to the soma in a palmitoylation-dependent manner to induce neurodegeneration. (**C**) Also in healthy axons, the labile, pro-survival factors NMNAT2 and SCG10 are continually supplied on axon transport vesicles into distal axons in a palmitoylation-dependent manner. (**D**) Following axonal injury, the constant supply of SCG10 and NMNAT2 from the soma into distal axons is blocked, and SCG10 and NMNAT2 are quickly degraded, inducing Wallerian degeneration. Figure created with BioRender.com. Abbreviation: NMNAT2, nicotinamide mononucleotide adenylyltransferase 2; SCG10, superior cervical ganglion 10.

In addition to its roles in neuronal development and presynaptic protein targeting and function, palmitoylation is a critical regulator of axon-to-soma signaling, axon integrity, and axon degeneration. Axotomy in cultured neurons or nerve transection or nerve crush *in vivo* are models of traumatic axon injury that activate intrinsic axon degeneration pathways both proximal and distal to the injury site. Palmitoylation of dual leucine-zipper kinase (DLK; also known as mitogen-activated protein kinase kinase kinase 12 [MAP3K12]) plays a critical role in axon injury pro-degeneration response in the proximal axon and soma [164,165]. Axon injury induces DLK-dependent axon and soma degeneration in sensory and motor neurons. DLK activation triggers an MAPK cascade involving its direct MAP2K targets MKK4 and MKK7 and the MAPK JNK, resulting in phosphorylation of the transcription factor c-Jun [166–168]. When DLK is palmitoylated, it localises to axonal transport vesicles where it complexes with JIP3, MKK4, MKK7, and JNK3 to allow retrograde pro-degenerative signaling in response to injury (Figure 2A,B) [164,165,169]. Blocking palmitoylation of DLK completely prevents JNK activation and degeneration both in cultured neurons and *in vivo* [164,165]. Indeed, blocking palmitoylation of JNK3 also blocks pro-degenerative phosphorylation of c-Jun following axonal injury [169]. These findings highlight the critical role palmitoylation plays in neurodegeneration and provide a strong rationale for identifying compounds that inhibit DLK palmitoylation to treat chronic neurodegeneration and acute neuronal injury [170].

Conversely, palmitoylation also regulates two important axon survival factors critical for Wallerian degeneration [140]. Wallerian degeneration is the degeneration of the axon distal to the injury site and is important in the peripheral nervous system to clear axonal debris to promote regeneration. However, ‘Wallerian-like’ degeneration is also observed in chronic degenerative conditions, so there is considerable interest in the mechanisms underlying this type of degeneration [136,171,172]. The microtubule-stabilising protein superior cervical ganglion 10 (SCG10; also known as stathmin-2 [STMN2]) is a short-lived protein that is constantly supplied to the axon by fast axonal transport and is implicated in TDP-43 neuropathies, in particular ALS [173]. Rapid degradation of SCG10 in healthy and injured axons is JNK dependent. Knockdown of SCG10 accelerates degeneration, whereas blocking JNK-dependent phosphorylation and subsequent degradation of SCG10 delays axon degeneration [174]. SCG10 is palmitoylated and transported anterogradely in axons in a palmitoylation-dependent manner by fast axonal transport (Figure 2C) [175,176]. Interestingly, its degradation also depends on palmitoylation and the activation of DLK/JNK [175]. These findings suggest that palmitoylation tethers SCG10 to transport vesicles to maintain a constant supply in distal axons but also plays a role in its labile nature (Figure 2C,D). This means that rate-limiting amounts of SCG10 are maintained in distal axons such that in the event of an axonal injury, SCG10 levels fall quickly so Wallerian degeneration can occur.

Nicotinamide mononucleotide adenylyltransferase 2 (NMNAT2) is another short-lived survival factor (half-life <1 h in cultured neurons). NMNAT2 is a nicotinamide adenine dinucleotide (NAD^+^)-synthesising enzyme that must be constantly supplied to the axon as a limiting factor for axon survival [177–179]. NMNAT2 knockdown causes axon degeneration in the absence of injury and, in injured neurons, NMNAT2 levels in the distal axon drop rapidly, inducing Wallerian degeneration [180]. This degeneration can be prevented by overexpression of NMNAT2 or expression of Wld^S^ (a stable, partially cytosolic form of NMNAT1, a paralogue of NMNAT2) that substitutes for NMNAT2 [177,178]. NMNAT2 is palmitoylated and transported anterogradely in axons in a palmitoylation-dependent manner by fast axonal transport (Figure 2C) [181,182]. However, palmitoylation-deficient NMNAT2 is less ubiquitinated, more stable, and, subsequently, more protective against axon degeneration when overexpressed in cultured neurons than wildtype palmitoylated NMNAT2 [182]. Interestingly, the DiAntonio group recently found that different populations of NMNAT2 are targeted for degradation by different mechanisms [175,178]. Palmitoylated NMNAT2 degradation is DLK/JNK dependent, whereas non-palmitoylated NMNAT2 degradation is dependent on the Phr1/Skp1a/Fbxo45 E3 ligase complex [175,178]. Like SCG10, palmitoylation of NMNAT2 likely provides a mechanism for its delivery over long distances to distal axons while also contributing to its labile nature to ensure it is present in distal axons in rate-limiting amounts to induce Wallerian degeneration in the event of an injury (Figure 2D).

Until recently, the PATs that mediate NMNAT2 and DLK palmitoylation in neurons were unknown. Interestingly, the Thomas group identified ZDHHC17 as the enzyme responsible for palmitoylation of both NMNAT2 and DLK [165]. Acute loss of ZDHHC17 blocks DLK-dependent pro-degenerative signaling and the induction of apoptosis following injury but the long-term loss of ZDHHC17 results in NMNAT2-dependent degeneration of distal axons. This finding is intriguing, but why would the same PAT be used to palmitoylate pro-degenerative and pro-survival proteins? It was hypothesised that palmitoylation of DLK and NMNAT2 by ZDHHC17, localised to the Golgi, forms a ‘trust but verify’ system. In this system, palmitoyl-NMNAT2 and palmitoyl-DLK are delivered to distal axons where palmitoyl-NMNAT2 will serve a neuroprotective role and palmitoyl-DLK is present to respond to axonal injury (Figure 2). Additionally, the regulation of NMNAT2 and DLK by a single PAT is advantageous as it allows for coordinated regulation of expression and function of DLK and NMNAT2 in the distal axon [165].

The DLK pro-degenerative signaling complex is not the only retrogradely trafficked complex regulated by palmitoylation. The palmitoylated glycoprotein 130 (Gp130, gene name *IL6ST*) is a critical component of another retrograde signaling complex that plays a role in neurogenesis, stem cell fate, and response to injury [56]. Gp130 receptor complexes respond to neuropoietic cytokines, including ciliary neurotrophic factor (CNTF), cardiotrophin-1, and leukemia inhibitory factor, to activate the Janus kinase/signal transducer and activator of transcription (JAK/STAT) signal transduction pathway [183]. Knockdown of axonal PATs ZDHHC5 and ZDHHC8 decreases Gp130 palmitoylation and surface expression as well as significantly attenuates retrograde signaling in DRG neurons by the Gp130/JAK/STAT3 pathway in response to CNTF stimulation [56]. Interestingly, JAK1 is also palmitoylated by ZDHHC3 and ZDHHC7, and its palmitoylation is required for downstream phosphorylation of STAT3 and neuronal survival [184].

## Palmitoylation-dependent AIS protein targeting

**Figure 3:**
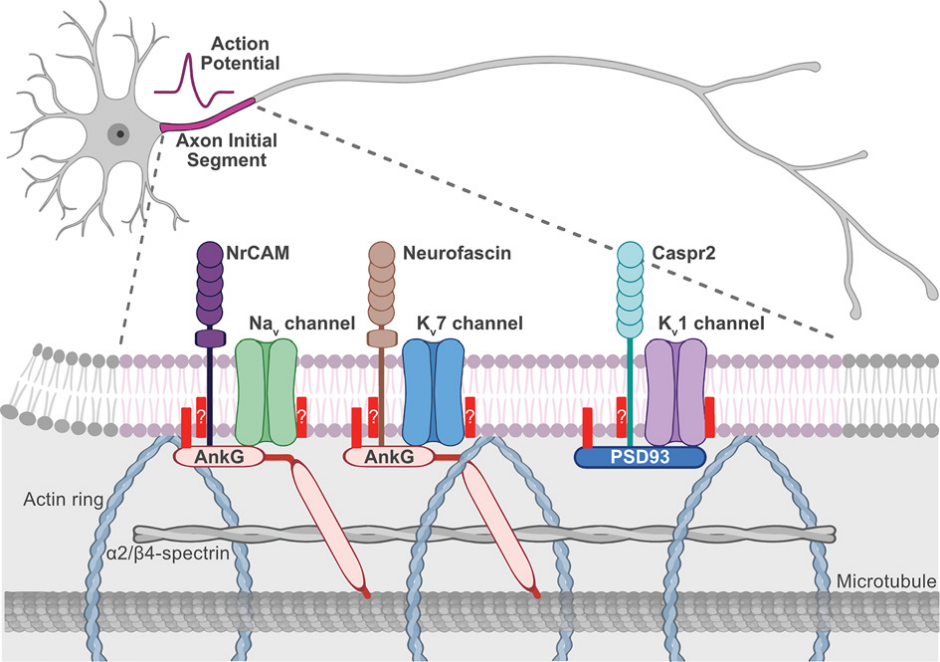
Palmitoylation-dependent regulation of protein targeting to the AIS The AIS is in the proximal part of the axon (pink) and is the site of action potential generation. Action potential firing is dependent on the high density of voltage-gated sodium (Na_v_; green) and potassium channels (K_v_7 [light blue] and K_v_1 [light purple]) at this site. These ion channels are clustered at the AIS via interactions with their scaffolds AnkG (light red) and PSD93 (dark blue) that also interact with the cell adhesion molecules NrCAM (dark purple), Neurofascin (brown), and Capsr2 (teal) and with the underlying actin, α2/β4-spectrin, and microtubule cytoskeleton. All these AIS components are either targeted to the AIS in a palmitoylation-dependent manner (solid red bar, AnkG, PSD93, and K_v_1 channels) or are palmitoylated, but the role of palmitoylation in AIS targeting is currently unknown (red bar with a question mark). Figure created with BioRender.com. Abbreviation: NrCAM, neuronal cell adhesion molecule.

An exciting new role for palmitoylation-dependent regulation of protein trafficking and targeting to another site within axons, the AIS, has recently emerged. The AIS is proximal to the soma and is the site of integration of synaptic input and generation of action potentials (Figure 3). The scaffold protein Ankyrin G (AnkG; ANK3) is highly enriched in the submembrane at this site along with voltage-gated sodium (Na_v_1) and potassium channels (K_v_7, and K_v_1) [185–188]. Anchoring of Na_v_1 and K_v_7 channels at the AIS critically requires AnkG [189–192]. Interestingly, AnkG is palmitoylated on a cysteine residue in the membrane-binding domain, which is required for its membrane association and polarisation in epithelial cells and its targeting to and scaffolding ability at the AIS in neurons (Figure 3) [193,194]. This was the first example of palmitoylation-dependent regulation of protein targeting at the AIS. AnkG AIS-binding partners Na_v_1 and K_v_7 channels are known or are predicted to be palmitoylated as well [18,195,196]. Whether or not palmitoylation plays a direct role in their AIS clustering is unknown, but this would be an interesting area for future investigation.

A subset of voltage-gated potassium channels, K_v_1.1, K_v_1.2, and K_v_1.4, are also present at the AIS but lack an AnkG-binding motif and do not associate with AnkG. How these proteins are targeted to the AIS has remained poorly understood [197,198]. One mechanism aiding in K_v_1 channel localisation to the AIS is through the association with the scaffold protein PSD93 [199,200]. PSD93 is a well-known palmitoyl protein, but until recently, the function of its palmitoylation was unclear [41,201,202]. PSD93 localises to postsynaptic sites, but palmitoylation is not required for its synaptic targeting [41]. Of the MaGUK family, PSD93 is the only one that enriches at the AIS [199] and, interestingly, its palmitoylation is required for its clustering at this site (Figure 3) [69].

The only PAT that contains a C-terminal type-I PDZ ligand (S/T-X-V) that would be predicted to bind to PSD93’s type-I PDZ domains is ZDHHC14 [68]. Indeed, the C-terminal PDZ ligand of ZDHHC14 binds PSD93’s third PDZ domain. The subsequent palmitoylation of PSD93 by ZDHHC14 also requires ZDHHC14’s PDZ ligand, suggesting that the ligand is important not only for PSD93 binding, but also for substrate recognition [69]. Indeed, ZDHHC14 is the predominant PSD93 PAT in neurons where loss of ZDHHC14 significantly reduces PSD93 palmitoylation and targeting to the AIS [69]. Consistent with this, expression of a palmitoylation deficient PSD93 in neurons also reduces PSD93 targeting to the AIS, albeit less intensely than with loss of ZDHHC14.

ZDHHC14 is a very interesting PAT and very little is known regarding its role in mammalian cells. It is highly expressed in the hippocampus [203,204] and is intolerant to loss of function genetic mutations in humans [205] and may play a role in cancers [206,207]. There were no known unique ZDHHC14 substrates prior to the identification of PSD93. One study identified the human β2-adrenergic receptor (β2AR) as a redundant substrate of ZDHHC9, ZDHHC14, and ZDHHC18. However, the specific contribution of ZDHHC14 to β2AR palmitoylation, particularly in a relevant cell type without overexpression is unclear [208].

At the AIS, PSD93 acts as a scaffold for K_v_1 channels [199]. K_v_1 clustering likely involves binding of the second PDZ domain of PSD93 to the C-terminal type-I PDZ ligands of K_v_1.1/1.2/1.4 [200,209]. K_v_1 channels are also palmitoylated on a cysteine residue in a highly conserved region in the loop between their second and third transmembrane domains [69,210]. Interestingly, the developmental expression of ZDHHC14 mirrors that of PSD93 and K_v_1.1/1.2/1.4 with expression first detectable at day *in vitro* (DIV) eight and increasing to DIV16, suggesting a shared function in hippocampal neurons. Indeed, palmitoylation of K_v_1 channels is also ZDHHC14-dependent. Furthermore, ZDHHC14 also regulates AIS targeting of K_v_1 channels themselves, such that loss of ZDHHC14 reduces AIS targeting of K_v_1 channels [69]. Like many other PATs, ZDHHC14 is predominantly localised to the ER and Golgi apparatus [55,56,69] but has also been observed on the plasma membrane in polarised endothelial cells [193] and on dendritic punctate structures and in axons, but not enriched at the AIS, in hippocampal neurons [69]. This suggests that palmitoylation of PSD93 and K_v_1 channels likely occurs early in their trafficking route prior to insertion in the AIS. However, these studies used overexpressed epitope-tagged ZDHHC14, so the true endogenous localisation and site of K_v_1/PSD93 palmitoylation are still unclear.

Intriguingly, ZDHHC14-deficient hippocampal neurons are hyperexcitable with a dramatic decrease in the density of outward currents as well as increased action potential firing [69]. These neurophysiological changes are consistent with a loss of K_v_1 channels at the AIS, although the effects of loss of ZDHHC14 on neuronal excitability are more dramatic than changes in AIS targeting of any individual K_v_1 channel [69]. This raises the intriguing possibility that ZDHHC14 may be responsible for palmitoylating other AIS components that may be contributing to the hyperexcitability phenotype.

Although loss of ZDHHC14 significantly reduces palmitoylation of PSD93 and K_v_1 channels, it does not entirely abolish palmitoylation. Another study suggested that ZDHHC17 palmitoylates the zebrafish orthologue of K_v_1.1, but the effects of endogenous neuronal K_v_1.1 after loss of ZDHHC17 were not examined [211]. Additionally, ZDHHC17 lacks a Type I-PDZ ligand, making it less likely to mediate residual PSD93 palmitoylation and, indeed, loss of ZDHHC17 *in vivo* in mice does not alter PSD93 palmitoylation [212]. However, whether other PATs contribute to palmitoylation of PSD93/K_v_1 channels is possible and warrants further investigation.

In addition to palmitoylation-dependent regulation of AIS targeting of AnkG, PSD93, and K_v_1 channels, we noticed that several other main AIS components are known to be palmitoylated or have been identified in palmitoyl proteomic studies, including Na_v_ and K_v_7 channels, as mentioned above, and AIS cell adhesion molecules neurofascin (NF186), neuronal cell adhesion molecule (NrCAM), and contactin-associated protein-like 2 (Caspr2; Figure 3). NF186 is palmitoylated and its palmitoylation is required for sorting to specialised membrane domains but not for AnkG binding, but whether palmitoylation regulates its AIS targeting is not clear [213]. NrCAM has been identified in one palmitoyl proteome [18] and has a cysteine in a homologous sequence to that of NF186 [213], so it is likely also palmitoylated, but no follow-up studies have been performed. Caspr2 has also been identified in one palmitoyl proteome study [18], but again, no follow-up studies have been performed.

To further understand the importance of palmitoylation in regulating the clustering and function of AIS proteins, we sought to determine whether palmitoylation is a prominent modification in the AIS proteome. Even though many individual proteins are well-known to be localised to the AIS, until recently there had been no systematic studies to define the entire AIS proteome [186]. However, a recent study from the Rasband group used BioID (multiplexed proximity biotinylation followed by mass spectrometry) to map proteins localised to the AIS [214,215]. They used BirA* biotin ligase fused to NF186, Ndel1, and Trim46 to identify the AIS membrane-proximal, shallow cytoplasm, and microtubule-associated proteins, respectively.

Using the proteins that showed the most significant change between control and experimental conditions (i.e., Figure 2A and Figure 7F, and Supplemental Figure 6B of [214]; see reference for details), we compiled a single, non-redundant list of putative AIS proteins. The BioID study did not identify several known AIS proteins, including Na_v_1.6, K_v_1 channels, Na_v_ and K_v_1 β subunits, PSD93, K_v_7.2, K_v_7.3, NrCAM, and Caspr2. We manually added these proteins to the NF186 proteome and obtained a cumulative AIS proteome dataset comprised of 172 genes (Supplementary Table S1). This dataset was then uploaded to the palmitoyl protein database SwissPalm and compared with all available human, mouse, and rat palmitoyl proteomic datasets (54 in total) [18]. Due to the rat nomenclature of the proteins used in the BioID dataset, some were not recognised in the palmitoyl-proteome comparison, so all negative hits in the AIS dataset, i.e., not found in any study or found in only one palmitoyl proteome, were screened manually in SwissPalm. All proteins/orthologues identified in at least two palmitoyl proteomic studies or one targeted study were counted as palmitoylated. As a control comparison, the synaptic gene set (SynGO release 1.1, all genes) was downloaded from the SynGO database and subjected to the same comparison using SwissPalm [18,216].

**Figure 4:**
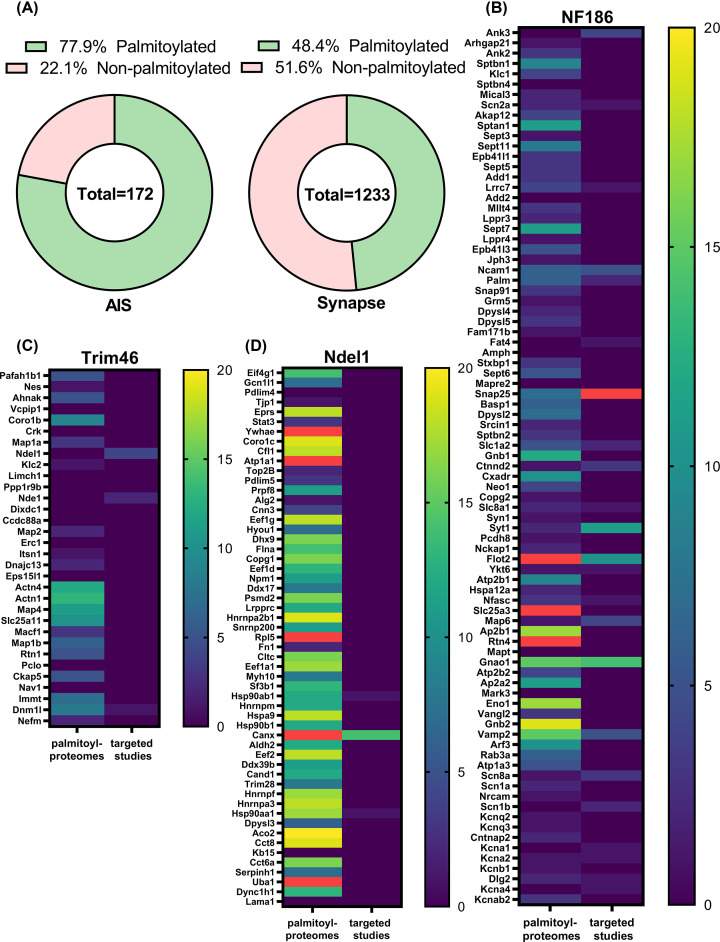
Palmitoyl-proteome comparison analysis reveals the prevalence of palmitoylation among AIS proteins (**A**) Donut charts showing the percentage of palmitoylated proteins in AIS or synaptic proteomes. The totals indicate the number of proteins in each dataset. The AIS proteome gene list was assembled from the highest confidence AIS proteins identified by Hamdan et al*.* ([214]) and several known AIS proteins missed in the Hamdan study. Synaptic proteome genes were extracted from the SynGO database (SynGO release 1.1-all genes [216]). Both gene sets were subjected to palmitoyl-proteome comparison analysis in SwissPalm using all 54 available mouse, human, and rat proteomes. All proteins found in at least two palmitoyl proteomes or one targeted study were designated as palmitoylated. (**B–D**) Heat maps showing all genes in membrane-proximal (NF186, B), microtubule-associated (Trim46, C), and shallow cytoplasm (Ndel1, D) and AIS proteomes and the number of palmitoyl proteome and targeted studies for each gene. Red colour designates values above 20. The data on the number of studies were extracted from the palmitoyl-proteome comparison analysis described above.

Surprisingly, our new analysis revealed that a staggering 77.9% of the AIS proteome is likely to be palmitoylated (Figure 4A, Supplementary Table S2). This was much higher than the 48.4% of palmitoylated proteins at the synapse, a well-known palmitoylation hotspot (Figure 4A) [17,20]. Further analysis of palmitoylation in each proteome revealed that low-throughput targeted studies frequently verified proteins identified in the NF186 proteome, but some were only rarely found in high-throughput palmitoyl proteomic studies (Figure 4B). In contrast, those proteins identified in the Trim46 (microtubule-associated) proteome were commonly identified in high-throughput palmitoyl proteomic studies, but rarely verified by low-throughput targeted studies (Figure 4D). The Ndel1 (shallow cytoplasm) proteome was the least enriched for palmitoylated proteins (Figure 4C).

The NF186 proteome contained the highest number of known palmitoylated AIS proteins, such as AnkG, NF186, and Na_v_1.6 [193,196,213]. It was also the proteome most enriched in low-throughput targeted verification studies. These observations indicate that palmitoylation of the AIS membrane-proximal proteome is the most well-verified and likely to be highly significant to the function of the AIS. Proteins identified by Trim46, despite showing a high enrichment in high-throughput palmitoyl proteomic studies, have been largely missed by low-throughput targeted investigations (except for calnexin). This proteome contains several families of proteins, such as ribonuclear proteins, chaperones, chaperonins, and Dihydropiriminidase-related proteins, which have been previously unknown to localise to the AIS or to be verified as palmitoylated. Given these observations, one promising avenue for further investigation would be to study palmitoylation of AIS microtubule-associated and cytoplasmic proteins.

## Conclusions and future directions

The bulk analysis of the AIS proteome for palmitoylated proteins reveals that palmitoylation is likely to be a crucial modification of AIS proteins, even more so than at the synapse. Our review provides an extensive basis for follow-up study. Indeed, the only AIS proteins known to localise to the AIS in a palmitoylation-dependent manner are AnkG, PSD93, and K_v_1 channels. The vast enrichment of the AIS proteome for palmitoylated proteins is an intriguing finding and may suggest that palmitoylation provides a broad mechanism to anchor AIS-resident proteins at this site. Indeed, the AIS is a very structurally dense subcellular compartment (i.e., high density of proteins localised to a relatively small and narrow area) that acts as a trafficking gateway to the rest of the axon [217–219]. Thus, it is plausible that in such a dense environment with a lot of cargo passing by firm attachment and anchorage to the AIS membrane is provided by palmitoylation for many AIS proteins. Alternatively, palmitoylation could be a mechanism that is required for tethering proteins to transport vesicles or motor protein subunits and/or interaction with scaffold proteins during trafficking to the AIS [164,220].

Though our new analysis and the studies discussed here suggest that palmitoylation may participate in trafficking and retention of proteins at the AIS, several questions remain unanswered in the current literature. Is palmitoylation necessary and/or sufficient for AIS targeting or do protein–protein interactions dominate in determining AIS targeting? Are palmitoylated AIS proteins modified with a particular fatty acid that is preferentially inserted into the AIS plasma membrane? Is there a unique aspect about the number or position of acyl modifications that serves as the trafficking/retention signal? Even in the case of K_v_1 channels and PSD93 it is unknown if palmitoylation of one or both are required for their interaction and whether this interaction occurs on the Golgi membrane or if PSD93 captures and retains K_V_1 channels at the AIS. Answering these questions will be crucial for identifying the factors that determine how palmitoylation participates in establishing and organising axonal subcompartments.

## Supplementary Material

Supplementary Tables S1-S2

## Data Availability

All associated datasets are available as supplementary data files.

## Abbreviations

ABHD: α/β hydrolase domain-containing protein
AD: Alzheimer disease
AIS: axon initial segment
ALS: amyotrophic lateral sclerosis
AnkG: Ankyrin G
APH-1: anterior pharynx-defective 1
APP: amyloid precursor protein
APT: acyl protein thioesterase
Aβ: β-amyloid
BACE1: β-site APP cleaving enzyme 1
Caspr2: contactin-associated protein-like 2
cGMP: cyclic guanosine monophosphate
CNTF: ciliary neurotrophic factor
CNP: C-type natriuretic peptide
CRD: cysteine-rich domain
DLK: dual leucine-zipper kinase
DRG: dorsal root ganglia
GABA: γ-aminobutyric acid
GAD65: glutamate decarboxylase 65
Gp130: glycoprotein 130
HD: Huntington disease
HTT: huntingtin
JAK/STAT: Janus kinase/signal transducer and activator of transcription
JNK: c-Jun N-terminal kinase
MaGUK: membrane-associated guanylate kinase
MAP6: microtubule-associated protein 6
NCAM: neural cell adhesion molecule
NMNAT2: nicotinamide mononucleotide adenylyltransferase 2
NrCAM: neuronal cell adhesion molecule
PAT: palmitoyl acyltransferase
PD: Parkinson disease
PDZ: C-terminal PSD95/discs large/ZO-1
PPT: palmitoyl protein thioesterase
PSD93: postsynaptic density 93/chapsyn 110/DLG2
SCG10: superior cervical ganglion 10
SNAP25: synaptosome-associated protein of 25 kDa
SNARE: synaptic soluble *N*-ethylmaleimide-sensitive fusion attachment protein receptor
SOD1: superoxide dismutase 1
VAMP: vesicle-associated membrane protein
β2AR: β2-adrenergic receptor
